# Scaling parent management training through digital and microlearning approaches

**DOI:** 10.3389/fpsyg.2022.934665

**Published:** 2022-09-21

**Authors:** David Grodberg, Irene Smith

**Affiliations:** ^1^Brightline Inc., Palo Alto, CA, United States; ^2^Brightline Medical Associates, Palo Alto, CA, United States

**Keywords:** parent management training (PMT), pediatric behavioral health care, digital mental health care, digital behavioral health care, parent training, digital parent training

## Introduction

While the return to school after the COVID-19 pandemic-related closures was welcomed by many kids and families, the transition back to “normal life” has also been hard as a result of profound social isolation and loneliness experienced by the overall population and financial distress for many (Echegaray, 2021; O'Sullivan et al., 2021). As a result, we are seeing an increase in mental health conditions and disruptive behaviors in children and adolescents at home and school. Rates of depression and anxiety among children have doubled since the pandemic began. At present, one in four children experience increased depression symptoms and one in five experience increased anxiety symptoms (Racine et al., 2021).

For families with children facing these issues, accessing care is an ever-growing problem. In total, 70% of counties in the United States do not have a child psychiatrist (McBain et al., 2019; Workforce Issues, 2019). This provider shortage coupled with long waitlists for both therapy and psychiatric services makes access to timely care difficult for many parents and caregivers struggling to effectively respond to challenging behaviors and mental health conditions. As a result, care may become reactive instead of proactive, and often, in the context of an acute crisis, interventions may focus on safety and stabilization as opposed to finding more long-term solutions (Waterman et al., 2015; Workforce Issues, 2019). Due to the lack of access and the high cost of care, many families are forced to wait until a crisis occurs and emergency care is needed—as evident by the 24% increase in mental health-related emergency room visits for children (ages 5–11) and the more than a 30% increase in mental health-related visits for adolescents (ages 12–17) in 2020 (Leeb et al., 2020; The White House, 2021). Similar to medication, emergency and urgent care are high-cost options that do not guarantee long-term, improved outcomes for the child.

Since relationships between children and their families and caregivers play a crucial role in children's emotional and mental wellbeing, involving parents in their child's behavioral health care is critical. Parents are the first line of defense for recognizing and managing their child's behavioral health needs and for promoting their wellbeing. Research suggests that with increased parental involvement in a child's mental and behavioral healthcare, the child may be 3× more likely to achieve better outcomes (Mbwana et al., 2009). One proven approach to involve parents and increase positive behavior in children is through parent management training (PMT) (Kazdin et al., 1992; Kazdin, 1997; Mabe et al., 2001). However, despite the success of PMT in decreasing aggressive, defiant, and oppositional behavior in children, most parents and caregivers do not have access to this evidence-based training. We aim to identify the barriers families face in accessing PMT and suggest approaches to scale this training model to ensure that PMT becomes an available opportunity for all families.

## Parent management training and its benefits

Parent management training is an intervention approach used to treat children, typically between the ages of 4 and 12, who display oppositional, aggressive, and antisocial behaviors, such as oppositional defiant disorder (ODD) and conduct disorder (Kazdin, 1997; Mabe et al., 2001; Diaz-Stransky et al., 2020). According to the American Academy of Pediatrics, PMT should be the first-line intervention for children under the age of 6 with ADHD or disruptive behaviors and can be coupled with medication for children over the age of 6 (Haine-Schlagel and Walsh, 2015). In PMT programs, parents learn behavioral management tools and techniques to effectively respond to their child's behavioral health needs. These techniques include identifying triggers or antecedents, understanding the child's response, and using the appropriate rewards or consequences to support behavior change at home.

Overall, PMT is beneficial to the child because it “positively affects parent-child relationships, mood, social competence, and school adjustment or performance” (Mabe et al., 2001). Parents learn positive communication pathways and techniques that promote children's development to their fullest potential. Evidence strongly suggests that changing parenting behaviors lead to improved behavioral health outcomes for children: PMT has a 92% success rate in decreasing aggression, defiance, and oppositional behavior for children with ODD, conduct disorder, disruptive mood dysregulation disorder, and intermittent explosive disorder (Kazdin, 2017). Furthermore, children whose families participate in PMT have improved prosocial behavior at school, and decreased oppositional and non-compliant behaviors (Sukhodolsky et al., 2016; Kazdin et al., 2018). Most children also saw long-term benefits such as decreased and/or discontinued coercive exchanges with parents and caregivers, along with improved academic performance, and increased emotional adjustment even after the training was completed (Long et al., 1994; Kazdin, 2008).

Research indicates that for children with ADHD, starting with PMT and then adding medication as needed improved behavior more than starting with medication and then adding PMT—with oppositional behavior decreasing by 50% from baseline observations (Pelham et al., 2016). Therefore, prioritizing parent training over medication can potentially lead to better outcomes for the child. Parents of children who began with behavioral therapy instead of medication also spent an average of $700 less annually because their child required a lower dose of medication or even no medication at all (Page et al., 2016; Rodden, 2016).

Outside of improved behavioral outcomes and decreased care costs, the benefits of PMT extend beyond the individual child. Parent training can often improve the relationship between parents and their children by creating positive communication pathways, and increasing self-awareness and self-management techniques. Parents tend to see improved stress levels, decreased depressive and anxiety symptoms, and increased perception of parenting competence, which can empower them to become better sponsors, allies, and role models in their child's care (Webster-Stratton and Herman, 2008; Kierfeld et al., 2013; Stattin et al., 2015; Colalillo and Johnston, 2016). The family unit as a whole also benefits from PMT through increased family resilience, improved sibling behavior, increased marital satisfaction, and better parental functioning (Bonin et al., 2011; Slusher, 2020).

## Barriers and criticisms of parent management training

Despite these many benefits, less than a third of parents have access to PMT training (Center for Disease Control, 2021). PMT training is traditionally delivered by therapists in in-person settings, thus introducing a number of access barriers. Barriers that prevent parents from accessing PMT include the location of training, transportation, costs, insurance coverage, scheduling, and childcare (Lundahl et al., 2006; Baker et al., 2011; Thornton and Calam, 2011; Diaz-Stransky et al., 2020; Weisenmuller and Hilton, 2021). And, even in cases where parents have access to PMT, upwards of 25% decline in enrollment, and between 26 and 51% do not complete treatment (Chacko et al., 2016; McCabe et al., 2020). This high level of declined enrollment and attrition is primarily a result of parental stress, lack of understanding of their children's mental health issues, and the logistical barriers to attendance noted above (Axelrad et al., 2013). Racial and socioeconomic status might further increase these numbers—research shows that black, indigenous, and people of color (BIPOC) families are less likely to participate in PMT than Caucasian families due to lower recruitment rates and socioeconomic factors (Axelrad et al., 2013; McCabe et al., 2020).

External factors are not the only barriers to parents accessing PMT. Barriers associated with stigma also keep parents from engaging with care—namely feelings of defensiveness, fear of being perceived as a bad parent, and feeling as though their child is being pathologized (Diaz-Stransky et al., 2020; Weisenmuller and Hilton, 2021). Failure to enroll or complete treatment can be associated with parents feeling discouraged or hopeless about the prospects of helping their child (Diaz-Stransky et al., 2020). Moreover, parents may also decline enrollment because of the perception that improved outcomes may decline or dissipate once training is over (Plessy, 2019). In addition to parental barriers, provider-specific barriers such as the need to serve large swaths of parents within time constraints contribute to the lack of large-scale dissemination of these interventions.

## Scaling parent management training

To address these barriers and criticisms and increase access, enrollment, and engagement, we recommend scaling PMT through digital, telehealth, and microlearning approaches. Evidence shows that utilizing telehealth increases access to care in underserved areas and is an effective approach to filling an unmet need for mental health services broadly and improving equity in access. Moreover, telehealth options have already been proven to mitigate barriers to care such as location, scheduling, transportation, and childcare, which can lead to improved adherence to parent training programs (Ollendick et al., 2016; Rooks-Ellis et al., 2020). Research has shown that digital parent training is beneficial because it allows care “to reach families in real time with best practice information [which] holds the potential to be a categorical shift in the ability to work effectively with families” (Macmillan, 2021). Digital parent training might also have an advantage over in-person PMT for engaging young parents, who prefer to access parent information and training online (Feil et al., 2018). The ease of accessibility and usability has been shown to lead to substantially increased completion rates of between 42% and 99%. Increased completion rates are directly correlated with improved long-term outcomes (Breitenstein et al., 2014). Thus, scaling parent training through digital and microlearning approaches can not only show improved outcomes for children experiencing disruptive behavioral health conditions but also decrease the need for face-to-face interventions with a mental health professional and alleviate an unmet demand for provider-led or intensive mental healthcare (Gao et al., 2020).

The digital PMT approaches can include a blend of self-guided, asynchronous content with synchronous coaching (Baumel et al., 2017; Diaz-Stransky et al., 2020). However, it is essential to provide these materials in a way that parents can access them quickly and easily throughout their day. Having already been implemented to promote self-care behaviors, and treat anxiety and depression, a microlearning approach to PMT can provide that quick and easy access that parents need (Wang et al., 2020; Zarshenas et al., 2020; Suffoletto et al., 2021). Microlearning is an approach to teaching and training that provides small, bite-sized pieces of content that only take 3–5 min to digest, such as videos, podcasts, multiple choice questions, and downloadable materials (Wang et al., 2020). Each burst of the content focuses on a specific learning outcome or goal and can provide quick, immediate answers to pertinent questions. Microlearning also allows parents to pick when and where they participate in training during their busy schedules. Supplementing other PMT digital care models, such as short sessions with a PMT coach, with microlearning approaches places the parent's preferences for receiving information and training at the forefront. Parents can find a blend between self-guided content, asynchronous discussion, and weekly coaching that fits their needs and schedule. This level of personalization also correlates to parental satisfaction which correlates to improved outcomes.

However, regardless of the mode of delivery, providers must encourage and educate parents and caregivers on the benefits of PMT to increase the likelihood that they will enroll and engage in training. It is also important that providers address parents' and caregivers' fears and concerns associated with PMT, such as feelings of defensiveness and stigma toward mental health treatment (Plessy, 2019). Providers should foreground that digital parent training is completed in the comfort and privacy of their own home or place of choosing, which has the potential to lessen parents' fears of being seen as bad parents or as being judged for engaging in mental health care.

## Final considerations

There are two final factors to consider when scaling PMT through digital and microlearning approaches: (1) Digital health platforms must scale these programs within the context of broader clinical services to be able to escalate care if/when clinically indicated; (2) When possible, the programs should be offered in a network of care that has partnerships with payors and/or employers to make these programs as affordable as possible for families.

While providing parent training digitally is a viable way to solve access and engagement issues, it must be offered within the context of broader clinical services/networks to escalate care when the severity and acuity of a child or adolescent's mental health condition increases. By including parent training within a larger network of providers and services, parents are given access to different levels of care based on the severity of their child's conditions and symptoms, which can include scaling up to therapy, medication management, and other supports like speech-language therapy (Froelich et al., 2002; Aldred et al., 2004; Aman et al., 2009; Meadan et al., 2009; Mohammadi et al., 2016; Roberts et al., 2019; Helander et al., 2022). This increased access ensures that the full spectrum of issues a child is facing are addressed and coordinated between providers.

Schools have been widely discussed as an option for delivering digitally-enabled PMT interventions. When PMT is school-based, it has the potential to be a stabilizing influence for families. However, thus far, these trainings have been reported as uncommon due to cost, expertise, space, and time constraints from schools. For schools who have invested in this area, lack of parent enrollment or completion has similarly been documented, and strategies to engage parents such as parent-teacher conferences have been largely unsuccessful, due to barriers similar to those of traditional care, namely, distance, sociocultural stigma, time, and perceptions of educators. Strategies such as providing childcare services during training, adopting a more collaborative approach in designing and delivering the training, and finally incorporating technology to improve ease of access (Ouellette and Wilkerson, 2008). As a means to effectively scale PMT within this setting, schools should partner with existing technology-based interventions that have the resources and expertise to provide this training. The combination of these approaches will improve adoption and enhance the potential to improve equity.

Another reason for offering programs within a larger network of care is the ability to partner with payors and employers to increase the affordability for families. While there is currently a lack of uniform coverage policies regarding which digital mental health care services are reimbursed and at what rate, within contexts that employ licensed clinicians and can offer diagnoses, there is an opportunity for payors and employers to help cover the costs of mental health care and programs (Ellimoottil, 2021; Hellman, 2022). In turn, this can reduce the out-of-pocket expenses for families and ultimately reduce the overall total care costs for payors due to increased mental health service utilization and improved outcomes.

## Conclusion

For many children, the return to school and “normalcy” has been a relief. However, high rates of reported psychosocial and behavioral problems in children have led to a subsequent surge in requests for pediatric mental and behavioral health services. Although it is a long-term goal to increase the number of pediatric mental health providers, that solution is not only too far off, but also not the only solution. Traditional behavioral health care options for children most often do not include the parents or caregivers, who play a vital role in children's lives and mental health. Knowing that working with caregivers decreases disruptive behavior and widens access to positive interventions, it is imperative to scale parent training in ways that make sense for the parents and caregivers—through digital platforms and microlearning approaches and through partnering with schools and health organizations where appropriate. Digital pediatric mental health care providers must include PMT as a tier of their larger teletherapy or digital coaching offerings because these interventions set up children and families with behavioral issues for long-term success and provide much-needed relief to an overburdened system.

## Author contributions

Both authors listed have made a substantial, direct, and intellectual contribution to the work and approved it for publication.

## Conflict of interest

Authors DG and IS are employed by Brightline Inc., and Brightline Medical Associates.

## Publisher's note

All claims expressed in this article are solely those of the authors and do not necessarily represent those of their affiliated organizations, or those of the publisher, the editors and the reviewers. Any product that may be evaluated in this article, or claim that may be made by its manufacturer, is not guaranteed or endorsed by the publisher.
